# Development of Nectin4/FAP-targeted CAR-T cells secreting IL-7, CCL19, and IL-12 for malignant solid tumors

**DOI:** 10.3389/fimmu.2022.958082

**Published:** 2022-11-21

**Authors:** Fanfan Li, Shuping Zhao, Cheng Wei, Yaodi Hu, Tianlong Xu, Xueyi Xin, Tingwei Zhu, Liting Shang, Shanwen Ke, Jiang Zhou, Xiaojun Xu, Yue Gao, Ai Zhao, Jimin Gao

**Affiliations:** ^1^ Key Laboratory of Laboratory Medicine, Ministry of Education, School of Laboratory Medicine and Life Science, Wenzhou Medical University, Wenzhou, China; ^2^ Department of Hematology, Wenzhou Key Laboratory of Hematology, The First Affiliated Hospital of Wenzhou Medical University, Wenzhou, China; ^3^ Medical Laboratory, Fenghua District People’s Hospital, Ningbo, China; ^4^ Department of Hematology, The Seventh Affiliated Hospital of Sun Yat-Sen University, Shenzhen, China; ^5^ Department of Geriatric, Affiliated Hangzhou First People's Hospital, Zhejiang University School of Medicine, Hangzhou, China; ^6^ Zhejiang Qixin Biotech, Wenzhou, China

**Keywords:** chimeric antigen receptor T cell, malignant solid tumors, cancer-associated fibroblasts, nectin cell adhesion molecule-4, fibroblast activation protein, interleukin-7, interleukin-12, CCL19

## Abstract

**Background:**

Chimeric antigen receptor T (CAR-T) cell therapy has made significant advances for hematological malignancies but encounters obstacles in the treatment of solid tumors mainly due to tumor immunosuppressive microenvironment.

**Methods:**

Immunohistochemistry analysis was performed to examine the cellular expression of nectin cell adhesion molecule-4 (Nectin4) and fibroblast activation protein (FAP) in a variety of malignant solid tumors. Then, we engineered the fourth-generation Nectin4-targeted CAR-T (Nectin4-7.19 CAR-T) and FAP-targeted CAR-T (FAP-12 CAR-T) cells to evaluate their safety and efficacy *in vitro* and *in vivo*.

**Results:**

In our study, we firstly demonstrated the aberrant overexpression of Nectin4 on both primary and metastatic solid tumors and FAP on cancer-associated fibroblasts. Then, we found that our fourth-generation Nectin4-7.19 CAR-T cells expressed IL-7 and CCL19 efficiently and exhibited superior proliferation, migration, and cytotoxicity compared to the second-generation Nectin4 CAR-T cells, while FAP-12 CAR-T cells exerted their ability of targeting both murine and human FAP effectively *in vitro*. In a fully immune-competent mouse model of metastatic colorectal cancer, lymphodepletion pretreated mice achieved complete remission with human Nectin4-targeted murine CAR-T (Nectin4 mCAR-T) cells. In the NSG mouse model of lung metastases, Nectin4-7.19 CAR-T cells eradicated metastatic tumors and prolonged survival in combination with FAP-12 CAR-T cells.

**Conclusions:**

These findings showed that Nectin4-7.19 CAR-T cells had potential therapeutic efficacy and exerted a synergistic role with FAP-12 CAR-T cells, further demonstrating that Nectin4 and FAP were able to serve as promising targets for safe and effective CAR-T therapy of malignant solid tumors.

## Introduction

In recent years, chimeric antigen receptor (CAR) technology has revolutionized cancer therapy, particularly in blood cancers (1–4). However, CAR-T therapy for malignant solid tumors remains challenging owing to tremendous phenotypic heterogeneity, inefficient proliferation and short persistence of CAR-T cells, and immunosuppressive microenvironment in tumor stroma where inhibitory checkpoints lead to T-cell dysfunction, factors like adenosine and reactive oxygen species inhibit T cells, immunosuppressive cells like regulatory T cells and myeloid-derived suppressor cells promote tumor growth and inhibit T-cell activity, and cancer-associated fibroblasts (CAFs) deposit extracellular matrix to limit T-cell penetration and recruit other immunosuppressive cells (5–9).

Nectin cell adhesion molecule 4 (Nectin4) is a type I transmembrane protein whose extracellular domain is composed of three Ig-like domains (V-C-C type), participating in the formation and maintenance of adhesion junctions together with cadherin. Nectin4 is ubiquitously expressed in human embryonic cells but hardly in normal adult tissues, while it is highly expressed on the surface of malignant solid tumors such as urothelial cancer, ovarian cancer, and melanoma, playing key roles in various aspects of tumor progression like proliferation, angiogenesis, epithelial-to-mesenchymal transition, metastasis, DNA repair, tumor relapse, and poor prognosis of these epithelial malignancies (10–13). Enfortumab vedotin, an antibody-conjugated drug targeting Nectin4, has shown unprecedented response rates in locally advanced or metastatic urothelial carcinoma with a tolerable safety in a phase I clinical trial (NCT02091999) and a phase II clinical trial (NCT03219333), and is undergoing phase III clinical trial (NCT03474107) to demonstrate a survival benefit (14–16). Thus, the U.S. Food and Drug Administration granted accelerated approval to Padcev (enfortumab vedotin—ejfv), a Nectin4-directed antibody and microtubule inhibitor conjugate, being indicated for the treatment of adult patients with locally advanced or metastatic urothelial cancer who had previously received a PD-1/PD-L1 inhibitor and a platinum-containing chemotherapy (17). While a growing number of studies have indicated that Nectin4 may be regarded as a potential target for cancer immunotherapy (18, 19), no study so far has reported the use of Nectin4-targeted CAR-T cells for clinical therapy of malignant solid tumors. Thus, our phase I study (NCT03932565) has been ongoing to examine the safety and feasibility of Nectin4-7.19 CAR-T cells in patients with Nectin4-positive malignant solid tumors.

CAFs are the major components of tumor-associated stroma, forming a highly tumorigenic and immunosuppressive microenvironment (20, 21). Fibroblast activation protein (FAP), a type II serine protease with dual specificity of dipeptidyl peptidase and gelatinase activities, is expressed on CAFs in a majority of malignant solid tumors but rarely on fibroblasts in normal tissues, making it an attractive immunotherapeutic target (22, 23).

Here, our study showed that Nectin4-7.19 CAR-T cells displayed significant anti-tumor activity *in vitro* and *in vivo* and were not likely to cause unacceptable on-target off-tumor toxicities. Furthermore, the combination of Nectin4-7.19 CAR-T cell therapy and FAP-12 CAR-T cell therapy exhibited synergistic anti-tumor effects and thus may be a promising double-pronged approach for patients with Nectin4-positive malignant solid tumors.

## Materials and methods

### Cell lines

The HEK-293T cell line was purchased from the American Type Culture Collection (Cat#ACS-4500); ABC-1, HT1376, and A549 cell lines were purchased from Cobioer (Nanjing, China); the MDA-MB-453 cell line was a gift from Dr. Haihua Gu (Wenzhou Medical University); and the MC38 cell line was a gift from Dr. Jindan Wang (Wenzhou Medical University). All cells were maintained in DMEM supplemented with 10% heat-inactivated FBS in 5% CO_2_ at 37°C. Then, ABC-1, A549, and MC38 cells were transduced with lentivirus encoding the *Firefly-Luciferase-GFP* gene to generate Luc. ABC-1, Luc. A549, and Luc. MC38 cells; MC38 cells were transduced with lentivirus encoding the *human Nectin4-Firefly-Luciferase-GFP* gene to generate hNectin4-Luc. MC38 cells; HEK-293T cells were respectively transduced with lentivirus encoding the *human FAP-Firefly-Luciferase-GFP* gene or the *murine FAP-Firefly-Luciferase-GFP* gene to generate hFAP-Luc. 293T or mFAP-Luc. 293T cells. All of these transduced cells were sorted by flow cytometry.

### Generation of CAR constructs and mCAR constructs

Nectin4 CAR consisted of a single-chain variable fragment (scFv) derived from an antibody (24, 25) against human Nectin4, a human CD8 leader signal, a human 4-1BB co-stimulatory domain, and a human CD3ζ activation domain (4), while Nectin4-7.19 CAR was a tandem construct encoding Nectin4 CAR, interleukin (IL)-7, and CCL19 with two 2A peptide sequence (26, 27). FAP CAR was constructed by an scFv derived from an anti-FAP antibody (28) with a human 4-1BB and CD3ζ, while FAP-12 CAR was constructed with FAP CAR and interleukin (IL)-12 by 2A polypeptide strategy. These CARs were cloned into the pLenti-vector to obtain the recombinant plasmids. To construct the human Nectin4-targeted second-generation murine CAR (Nectin4 mCAR), the anti-human Nectin4 scFv was fused with the murine CD8α hinge region and transmembrane, the murine intracellular domain of 4-1BB, and murine CD3ζ (29). Then, the mCAR was cloned into upstream of an IRES-GFP marker in the MSCV retroviral plasmid pMIGR1.

### T-cell isolation and transduction

To isolate human T cells, peripheral blood mononuclear cells were extracted from whole blood of healthy donors by Ficoll density gradient centrifugation. T cells were enriched with Dynabeads^®^ Human T-Activator CD3/CD28 (Thermo Fisher Scientific, USA) and followed by stimulation for 24–36 h in the X-Vivo medium (Lonza, CH) supplemented with 50 IU/ml recombinant human interleukin (IL)-2 (PeproTech, USA) and then transduced with the lentiviral particles at multiplicity of infection (MOI) = 40. Mouse T cells isolated from spleen and lymph nodes of C57BL/6 mice by the Pan T Cell Isolation Kit II (Miltenyi Biotec) were activated with Dynabeads^®^ Mouse T-Activator CD3/CD28 (Thermo Fisher Scientific) and recombinant murine IL-2 (ProSpec) for 48 h, and then infected with retroviral particles at MOI = 10.

### Flow cytometry

Expression of Nectin4 on the surface of tumor cells was detected by anti-human Nectin4 Alexa Fluor^®^ 647-conjugated antibody (Clone #337516, R&D Systems, USA); Nectin4 CAR and Nectin4-7.19 CAR expression was detected by the fusion protein of Nectin4 extracellular domain with streptavidin (Nectin4-streptavidin), and then followed by the anti-streptavidin antibody with PE fluorescein (Clone #3A20.2). Expression of FAP was detected by anti-human FAP PE (Clone #427819, Bio-techne); FAP CAR expression was detected by anti-mouse IgG (H+L), Biotinylated Antibody (Cat #14709, Cell Signaling Technology, USA) and followed by streptavidin-tagged APC. Then, CAR-T cells were collected and their phenotype was assessed with anti-human CD4 PE/Cy7 (Clone: OKT4, Dilution: 1/400), anti-human CD8a Pacific Blue™ (Clone: RPA-T8), anti-human CD45RO Alexa Fluor^®^ 488 (Clone: UCHL1), anti-human CD45RA APC (Clone: HI100), and anti-human CCR7 PerCp/Cy5.5 (Clone: G043H7). For analysis of immunological checkpoints, the following antibodies were used: anti-human TIM3 Alexa Fluor^®^ 647 (Clone: 7D3, BD Biosciences), anti-human LAG3 PE (Clone: T47-530, BD Biosciences, USA), anti-human PD-1 APC (Clone: EH12.2H7), and anti-human CTLA-4 APC (Clone: L3D10). mCAR-T cells were labeled with anti-mouse CD8 PE (Clone: 53-6.7) and anti-mouse CD4 PE (Clone: RM4-5, Mutiscience, Hangzhou, CN), fixed and permeabilized with the LIVE/DEAD Fixable Aqua Dead Cell Stain kit (Invitrogen, CN), and labeled with anti-mouse IFN-γ APC (Clone: XMG1.2, Dilution: 1/50, eBioscience, Wuhan, CN) for intracellular staining. All antibodies of brands not mentioned above were from BioLegend and dilutions not mentioned above were 1/200. The isotype-matched IgG1 was used as a negative control. Cells were analyzed by a FACS Aria IIFlow Cytometer (BD Biosciences). Data were analyzed with FlowJo 10 (FlowJo, USA).

### Cytokine secretion analysis

Enzyme-linked immunosorbent assay (ELISA) was used to quantify the concentration of cytokines and chemokines. Culture supernatant of CAR-T cells was collected and then detected by an IL-7 ELISA kit (Mutiscience) and a CCL19 ELISA kit (NeoBioscience, Shenzhen, CN), respectively.

### Proliferation analysis

CAR-T cells were labeled with CellTrace™ CFSE (Thermo Fisher Scientific) and co-cultured with tumor cells at an Effect/Target ratio of 1:1 in a 24-well plate without the addition of external cytokines for 5 days and then analyzed using a flow cytometer with 488-nm excitation and emission filters appropriate for fluorescein to assess the proliferation of CAR-T cells.

### Migration analysis

Chemotaxis on T cells was measured with a transwell (Corning, USA) with a 5-µm pore permeable membrane insert. Untransduced T cells labeled with CellTrace™ CFSE were added to the upper chamber of the transwell, and the 5-day CAR-T cell culture supernatant without any cytokines was collected and 400 μl was added to the lower chamber. After 2 h of incubation, untransduced T cells migrating into the lower chamber were observed with a fluorescence microscope and pictures from three horizons were taken at random (30).

### Cytotoxicity analysis

The xCELLigence RTCA MP instrument (Acea Biosciences Inc, CA, USA) was utilized for the assessment of CAR-T cell-mediated cytotoxicity (31). Briefly, 1 × 10^4^ tumor cells were seeded on each well of an E-Plate 16 (Acea Biosciences) and grew until their adherence. Then, CAR-T cells were added into each unit at different Effect/Target ratios, with media or 2.5% Triton-X 100 (Solarbio, Beijing, CN) as negative or positive controls. Each group consisted of three replicate wells and the impedance signals (Cell index) were recorded for a duration of 24–48 h. Electrical impedance was quantified every 15 min by the use of the RTCA DP Analyzer.

In the luciferase bioluminescence technique, tumor cells expressing luciferase reporter were plated into a 96-well plate (32). T cells were added with different Effect/Target ratios after target cells adhered onto the well. Media and 2.5% Triton-X 100 were regarded as a negative control (*K*_min_) and a positive control (*K*_max_), respectively. Each group consisted of three replicate wells. After 12 h co-incubation, cells were centrifuged and the supernatant was removed. Then 200 μl of serum-free DMEM medium containing 0.5 mM D-luciferin (MedChemExpress, Shanghai, CN) was added to each well, and the fluorescence intensity was measured by Luminometric Measurement on a microplate reader after 10 min. The fluorescence intensity value *K* of each well was counted, and the killing efficiency was equal to (*K*_min_ − *K*)/(*K*_min_ − *K*_max_) × 100%.

### Animal experiments

In the metastatic colorectal cancer model, the fully immune-competent male 6- to 8-week-old C57BL/6 mice (Charles River, Beijing, China) were inoculated subcutaneously (s.c.) at the right flank with 1.0 × 10^6^ hNectin4-Luc. MC38 cells on Day 0. To evaluate the dose dependence of Nectin4 mCAR-T cells, 5.0 × 10^6^ untransduced mouse T (mUTD) cells and different doses of hNectin4 mCAR-T cells were injected intravenously (i.v.) on Day 10 after randomization of mice (*N* = 6 mice per group). To improve the anti-tumor efficacy, cyclophosphamide (CPA) at 100 mg/kg was administered intraperitoneally (i.p.) 3 days before the infusion of 5.0 × 10^6^ mUTD or hNectin4 mCAR-T cells (*N* = 6 mice per group). The tumor volumes of the mice were recorded every 2 days and calculated as length × (width)^2^ × 0.5. In the metastatic lung cancer model, the severely immunodeficient male 6- to 8-week-old NSG mice (NOD-Prkdc^em26Cd52^Il2rg^em26Cd22^/Nju) (GemPharmatech Co, Ltd, Nanjing, China) were injected i.v. with 1.0 × 10^6^ Luc. ABC-1 cells on Day 0. After randomization (*N* = 3 mice per group), mice were treated i.v. with different doses of CAR-T cells on Day 7. Treatment with untransduced T (UTD) cells served as a negative control. Tumor progression was confirmed regularly by BLI using a Xenogen IVIS imaging system (PerkinElmer, Shanghai, CN), and the intensity of the signal was measured as total photon/second/cm^2^/steradian (p/s/cm^2^/sr). At the end of the experiment, mice were euthanized and tissues were resected for HE staining. All mice were bred and housed under SPF conditions in the Animal Center of Wenzhou Medical University. All mouse experiments were approved by the Laboratory Animal Ethics Committee of Wenzhou Medical University and performed in accordance with relevant institutions and national guidelines and regulations.

### Immunohistochemistry

Tumor tissues were obtained from patients at the Sixth Affiliated Hospital of Wenzhou Medical University to detect the expression of Nectin4 on tumor cells and FAP on CAFs. All informed consents were obtained from all included patients, and a supportive grant obtained from the Ethics Committee of the Sixth Affiliated Hospital of Wenzhou Medical University. For FAP staining, sections were blocked with 20% normal goat serum (Sigma, USA) in PBS for 30 min at room temperature and stained with 5 μg/ml primary mouse anti-human FAP antibody (Clone: EPR20021, Abcam) at 4°C overnight. The corresponding peritumoral normal tissues served as negative controls. The sections were rewarmed at 37°C for at least 45 min and incubated in the secondary antibody enhancement solution at room temperature for 20 min and then the secondary goat F(ab) anti-mouse IgG H&L (HRP) antibody (Abcam) at 37°C for 30 min. For Nectin4 staining, sections were stained with 10 μg/ml primary goat anti-human Nectin4, affinity-purified polyclonal antibody (Catalog # AF2659, R&D Systems) at 4°C overnight. Then, the sections were stained with the secondary biotinylated rabbit anti-goat IgG antibody (Abcam) at 37°C for 30 min and incubated in Streptavidin-Biotin Complex at 37°C for 30 min. Then, the sections were developed with SignalStain^®^ DAB Substrate Kit, counterstained with hematoxylin (Biocare Medical, Shanghai, CN) for 90 s, dehydrated with ethanol, clarified with xylene, and then examined under an optical microscope (Olympus, Japan).

### Statistical analysis

Data were analyzed as mean ± SD by *t*-test. Survival curve was analyzed by Kaplan–Meier curves and log-rank test. *p*-values < 0.05 were considered statistically significant. All experiments were repeated at least three times. All statistical analyses were performed with GraphPad Prism v6.0 (GraphPad Prism, USA).

## Results

### High expression of Nectin4 on malignant solid tumors and FAP on CAFs

**Figure 1:**
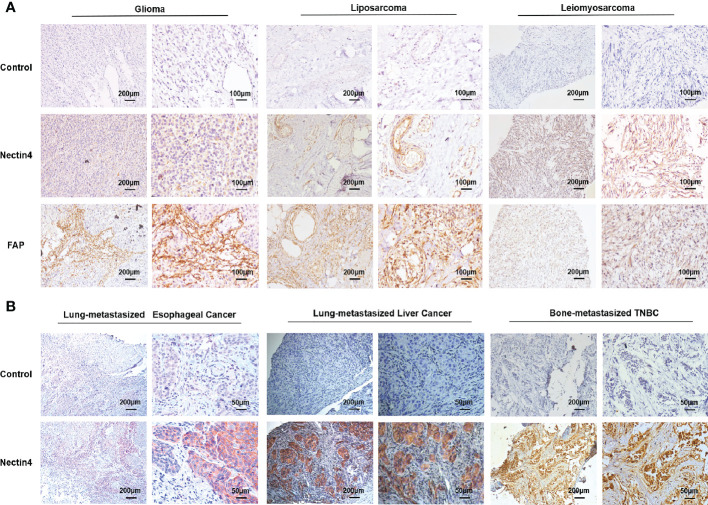
High expression of Nectin4 and FAP in a variety of cancers. **(A)** Expression of Nectin4 and FAP in glioma, liposarcoma, and leiomyosarcoma was assessed by IHC. **(B)** Expression of Nectin4 on lung-metastasized esophageal cancer, lung-metastasized liver cancer, and bone-metastasized triple-negative breast cancer (TNBC). Also see **Supplementary Figure 3**. Nectin4 is mainly located in the membrane (strongly positive) and cytoplasm (weakly positive) of cancer cells; FAP is mainly located in the membrane (strongly positive) and cytoplasm (weakly positive) of stromal cells, shown in brown.

Firstly, immunohistochemistry (IHC) analysis was performed to examine the cellular expression of Nectin4 in a variety of tumor biopsies. We found that not only common tumors such as lung, ovarian, and gastrointestinal cancers as previously reported (12, 24, 33–35), but also glioma, leiomyosarcoma, liposarcoma, gingival carcinoma, nasopharyngeal carcinoma, and laryngocarcinoma highly expressed Nectin4 (**Figure 1A** and **Figure S1**). Furthermore, Nectin4 was also overexpressed on metastatic cancers (**Figure 1B**), especially bone-metastasized triple-negative breast cancer (TNBC), which was without the expression of estrogen receptor, progesterone receptor and proto-oncogene Her-2, indicating that Nectin4 could be used as a good therapeutic target for both primary and metastatic tumors.

It has been shown that FAP is overexpressed in tumor-associated stromal cells of epithelial tumors and its expression is related to advanced stages, worse prognosis, and poor survival. We found that FAP was overexpressed not only on CAFs of epithelial cancers (**Figures S2 and S3**), but also on mesenchymal cells of sarcomas (**Figure 1A**).

### Nectin4-7.19 CAR-T cells exhibited superior proliferation and lower differentiation

**Figure 2:**
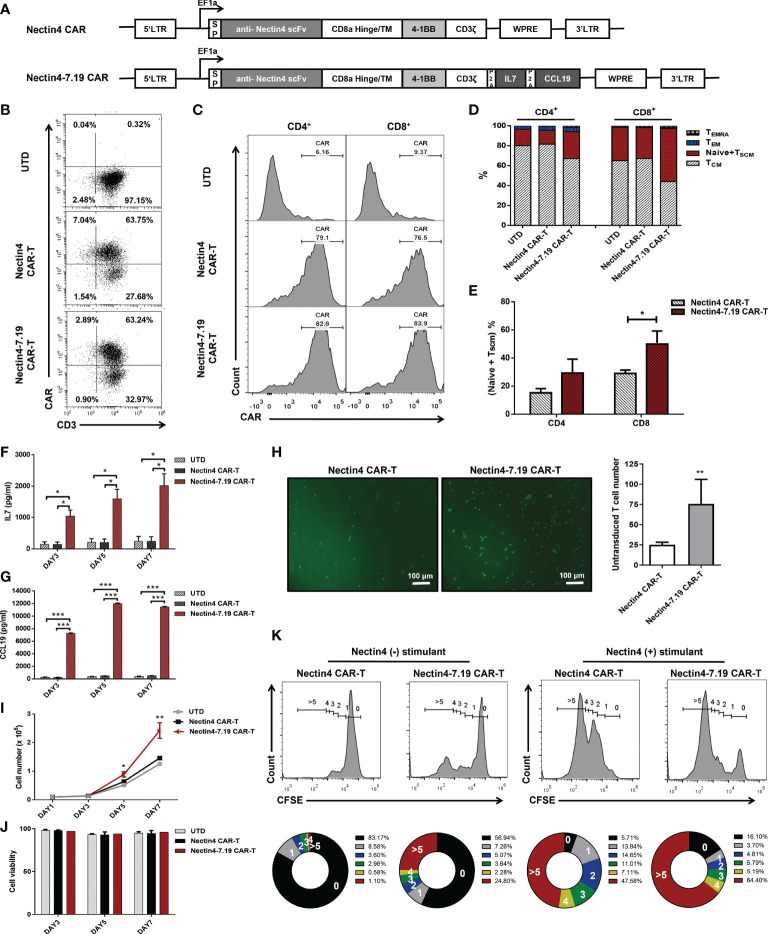
CAR structure and characterization of Nectin4-7.19 CAR-T cells. **(A)** Schematic illustration of Nectin4 CAR and Nectin4-7.19 CAR lentiviral vector. LTR: long terminal repeats; SP: CD8 signal peptide; TM: transmembrane region; P2A: 2A polypeptide element. **(B)** CAR expression in Nectin4 CAR-T and Nectin4-7.19 CAR-T cells was measured by flow cytometry. UTD indicates the untransduced T cells as a negative control. **(C)** Relative CAR expression in CD4^+^ and CD8^+^ T subsets. **(D)** Representative CAR-T cell phenotyping plot based on CD45RA and CCR7 in CD4^+^ and CD8^+^ T subsets. T memory stem cell, T_SCM_ (CD45RO^+^ CD45RA^+^ CCR7^+^); Naïve (CD45RO^-^ CD45RA^+^ CCR7^+^); central memory T cell, T_CM_ (CD45RO^+^ CD45RA^-^ CCR7^+^); effector memory T cell, T_EM_ (CD45RO^+^ CD45RA^-^ CCR7^-^); terminally differentiated effector memory T cell, T_EMRA_ (CD45RO^+^ CD45RA^+^ CCR7^-^). **(E)** Proportion of (Naïve + T_SCM_) subpopulation in CD4^+^ and CD8^+^ T subsets. **(F, G)** Secretion of IL-7 **(F)** and CCL19 **(G)** was examined by ELISA. **(H)** Transwell assays were performed to detect the chemotactic capacity of CFSE-labeled T cells after incubation with culture supernatant from Nectin4 CAR-T or Nectin4-7.19 CAR-T cells. Representative pictures (left panel) and statistical analysis diagram (right panel) are illustrated; scale bar = 100 μm. **(I, J)** Proliferative capacity **(I)** and viability **(J)** of Nectin4 CAR-T and Nectin4-7.19 CAR-T cells were tested by counting. **(K)** Epitope-driven proliferation for a comparison in Nectin4 CAR-T and Nectin4-7.19 CAR-T cells. Nectin4 CAR-T and Nectin4-7.19 CAR-T cells were labeled with CellTrace™ CFSE and co-cultured with ABC-1 cells (Nectin4^+^ stimulant) or 293T cells (Nectin4^−^stimulant) for 5 days in the absence of exogenous cytokines. The numbers of cell divisions are indicated in the histograms. The numbers in the donut charts represent percentages of each gated fraction in the cultured cells (0, black; 1, gray; 2, blue; 3, green; 4, yellow; and >5, red). Data represent the mean ± SD of three independent experiments; *p < 0.05, **p < 0.01, ***p < 0.001, *t*-test.

We constructed the human Nectin4-targeted second-generation CAR and fourth-generation CAR, designated Nectin4 CAR and Nectin4-7.19 CAR, respectively (**Figure 2A**). Flow cytometric analysis showed that the cell-surface expression of CAR in Nectin4-7.19 CAR-T cells was almost equivalent to that in Nectin4 CAR-T cells (**Figure 2B**). There was no significant difference in CAR expression between CD4^+^ and CD8^+^ T subsets in both Nectin4 CAR-T and Nectin4-7.19 CAR-T cells (**Figure 2C**). However, the proportion of the (Naïve + T_SCM_) subpopulation was higher in Nectin4-7.19 CAR-T cells than that in Nectin4 CAR-T cells, particularly in CD8^+^ T subsets (**Figures 2D, E**).

Then, we verified that Nectin4-7.19 CAR-T cells could produce IL-7 (**Figure 2F**) and CCL19 efficiently (**Figure 2G**). CCL19 secreted from Nectin4-7.19 CAR-T cells had chemotactic capacity to recruit more T cells (**Figure 2H**). As IL-7 has been shown to enhance the proliferation and viability of T cells (36), we investigated the absolute number and found that the proliferation of Nectin4-7.19 CAR-T cells was substantially stronger than that of Nectin4 CAR-T cells (**Figure 2I**), and their cell viability remained well (**Figure 2J**). Furthermore, after being stimulated by Nectin4-positive ABC-1 cells, Nectin4-7.19 CAR-T cells divided faster than Nectin4 CAR-T cells, indicating the specific antigen-driven proliferation (**Figure 2K**).

### Nectin4-7.19 CAR-T cells exhibited efficient cytotoxicity *in vitro*

**Figure 3:**
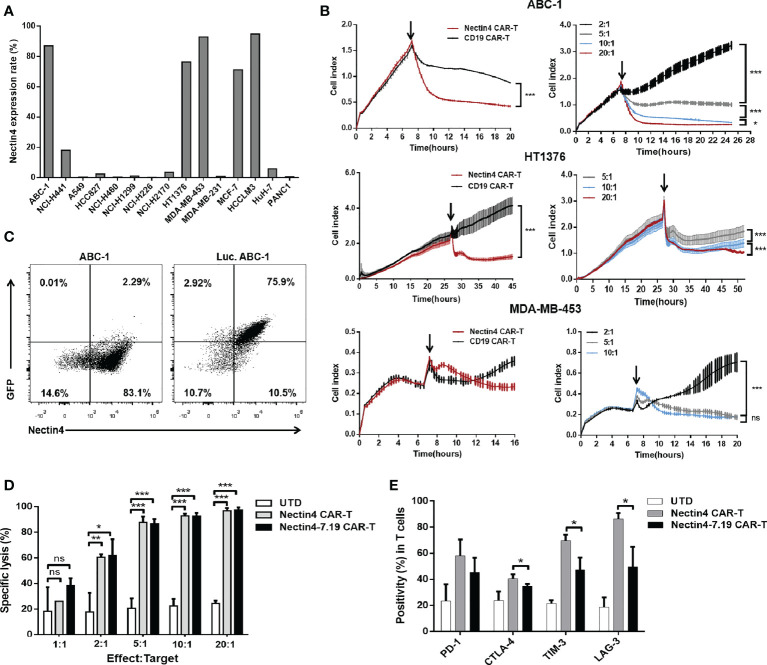
Efficient cytotoxicity of Nectin4-7.19 CAR-T cells *in vitro*. **(A)** Expression of Nectin4 on a panel of cancer cell lines. **(B)** Cytotoxicity of Nectin4 CAR-T cells against ABC-1, HT1376, and MDA-MB-453 cells was detected by xCELLigence RTCA label-free technology. The left panel compares the cytotoxicity between Nectin4 CAR-T and CD19 CAR-T cells against target cells at an Effect/Target ratio of 10:1; the right panel shows the killing efficacy of Nectin4 CAR-T cells at different Effect/Target ratios. Arrows refer to the addition of CAR-T cells. The *y*-axis is the normalized cell index generated by the RTCA software and displayed in real time to reflect the vitality of tumor cells. The *x*-axis is the time of cell culture in hours. **(C)** Nectin4 and GFP expression in Luc. ABC-1 cells transfected with lentivirus encoding the *Luciferase-T2A-GFP* gene. **(D)** Quantified data on the specific lytic levels of Nectin4 CAR-T and Nectin4-7.19 CAR-T cells against Luc. ABC-1 cells were assessed by luciferase bio-luminescence technique at different Effect/Target ratios *in vitro*. UTD served as a negative control. **(E)** Expression level of immune checkpoints was detected by flow cytometry after co-culture of Nectin4 CAR-T or Nectin4-7.19 CAR-T cells with ABC-1 cells at an Effect/Target ratio of 1:1 for 5 days. Data represent the mean ± SD of three independent experiments; ns, no significant difference, *p < 0.05, **p < 0.01, ***p < 0.001, *t*-test.

Flow cytometric analysis showed that high-level expression of Nectin4 was present on the surface of various tumor cell lines (**Figure 3A**). Then, we performed different assays to verify the specific cytotoxicity of Nectin4 CAR-T cells *in vitro* through the xCELLigence RTCA label-free technology and found that the co-incubation of Nectin4 CAR-T cells with ABC-1, HT1376, and MDA-MB-453 cells could cause an immediate and time-dependent decrease in cell index within 4 h, respectively, but not CD19 CAR-T cells (**Figure 3B**), demonstrating that Nectin4 CAR-T cells efficiently executed specific cytolysis against Nectin4-positive tumor cells and exhibited better cytotoxicity at the gradually increasing appropriate ratio of Effect/Target. To compare the cytotoxicity between Nectin4 CAR-T and Nectin4-7.19 CAR-T cells, we generated Luc. ABC-1 cells to express luciferase (**Figure 3C**) and observed that Nectin4 CAR-T and Nectin4-7.19 CAR-T cells exhibited equivalent oncolytic potentiality against Nectin4-positive Luc. ABC-1 cells (**Figure 3D**), but not Nectin4-negative Luc. A549 cells (**Figure S4**). Intriguingly, the expression of several immunological checkpoints on Nectin4-7.19 CAR-T cells were lower than those on Nectin4 CAR-T cells (**Figure 3E**).

### Nectin4 mCAR-T cells exerted anti-tumor effects on metastatic colorectal cancer in fully immune-competent mice

**Figure 4:**
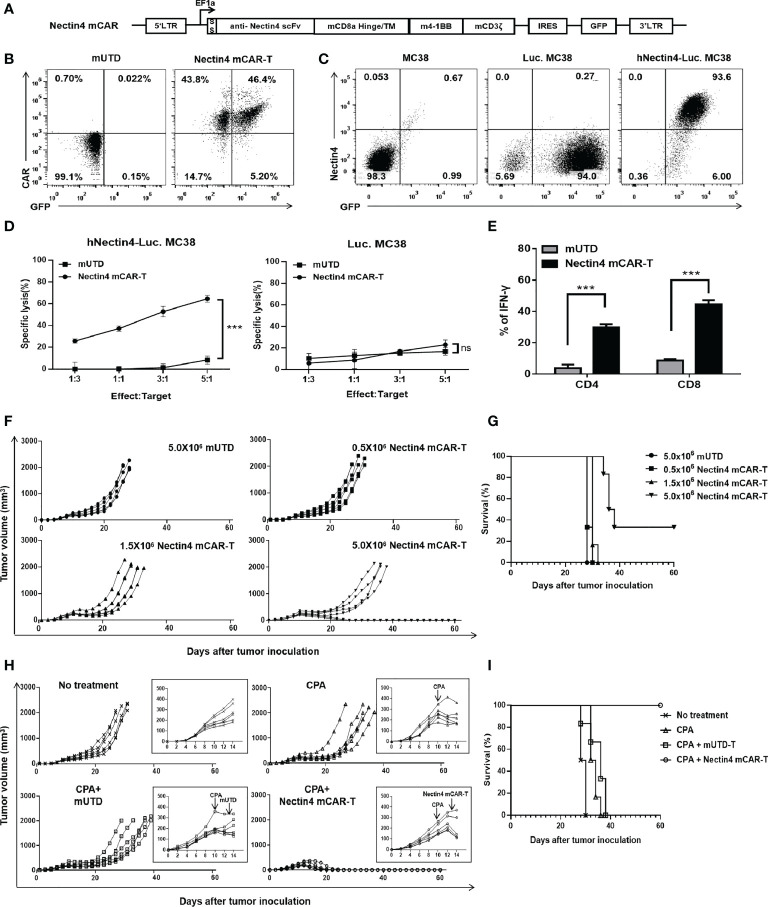
Therapeutic effect of Nectin4 mCAR-T cells on metastatic colorectal cancer in fully immune-competent mice. **(A)** The murine CAR construct was inserted upstream of an IRES-GFP marker in the MSCV retroviral plasmid pMIGR1. **(B)** mCAR expression of Nectin4 mCAR-T cells transfected with pMIGR1-mCAR-IRES-GFP retroviral particles. mUTD indicates the untransduced mouse T cells. **(C)** Nectin4 and GFP expression of Luc. MC38 cells and hNectin4-Luc. MC38 cells. **(D)** Quantified data on the specific lytic levels of Nectin4 mCAR-T cells against Luc. MC38 or hNectin4-Luc. MC38 cells were assessed by luciferase bio-luminescence technique at different Effect/Target ratios *in vitro*. ***p < 0.001, *t*-test. **(E)** Secretion of IFN-γ in CD4^+^ and CD8^+^ T subsets was assessed by flow cytometry after co-culture of Nectin4 mCAR-T cells or mUTD with hNectin4-Luc. MC38 cells for 12 h. ***p < 0.001, *t*-test. **(F, G)** C57BL/6 mice were s.c. inoculated with 1 × 10^6^ hNectin4-Luc. MC38 cells on Day 0 and injected i.v. with 5.0 × 10^5^ to 5.0 × 10^6^ Nectin4 mCAR-T cells on Day 10. A total of 5.0 × 10^6^ mUTD served as a negative control (*N* = 6 mice per group). Solid lines represent each individual mouse **(F)**. Kaplan–Meier survival curve is shown in **(G)**. *p*-values of log-rank tests were as follows: *p* = 0.35 (mUTD *vs*. 0.5 × 10^6^ Nectin4 mCAR-T); *p* = 0.09 (mUTD *vs*. 1.5 × 10^6^ Nectin4 mCAR-T); *p* = 0.0012 (mUTD *vs*. 5.0 × 10^6^ Nectin4 mCAR-T). **(H, I)** C57BL/6 mice were s.c. inoculated with 1.0 × 10^6^ hNectin4-Luc. MC38 cells on Day 0. Cyclophosphamide was i.p. administered at 100 mg/kg on Day 10 and 5.0 × 10^6^ Nectin4 mCAR-T cells or mUTD were i.v. injected on Day 13 (*N* = 6 mice per group). Solid lines represent each individual mouse **(H)**. Kaplan–Meier survival curve is shown in **(I)**. *p*-values of log-rank tests were as follows: *p* = 0.001 (CPA+Nectin4 mCAR-T *vs*. No treatment); *p* = 0.0006 (CPA+Nectin4 mCAR-T *vs*. CPA); *p* = 0.0008 (CPA+Nectin4 mCAR-T *vs*. CPA+mUTD).

Preclinical studies have been limited by the use of xenograft models that do not adequately recapitulate the immune system of a clinically relevant host, so we developed the Nectin4 mCAR-T cells to determine its anti-tumor effects in a fully immune-competent mouse model of metastatic colorectal cancer. Firstly, we constructed the mCAR with the anti-human Nectin4 scFv and used *pMIGR1-mCAR-IRES-GFP* retrovirus to transfect mouse T cells to prepare Nectin4 mCAR-T cells (**Figures 4A, B**). Then, we found that Nectin4 mCAR-T cells specifically recognized human Nectin4 and exhibited efficient cytotoxicity against hNectin4-Luc. MC38, but not Luc. MC38 cells (**Figures 4C, D**). Accordingly, the secretion of IFN-γ in CD4^+^ or CD8^+^ T subsets was higher in Nectin4 mCAR-T cells than those in mUTD cells (**Figure 4E** and **Figure S5**).

In order to explore the anti-tumor effect of Nectin4 mCAR-T therapy *in vivo*, C57BL/6 mice were subcutaneously inoculated with hNectin4-Luc. MC38 cells and treated with increasing doses of Nectin4 mCAR-T cells intravenously. Compared with the mice treated with mUTD cells, Nectin4 mCAR-T therapy at low dosage had no significant anti-tumor effect, but prolonged survival and even cured two mice without recurrence at high dosage (**Figures 4F, G**). For the purpose of improving survival, we then performed lymphodepletion with CPA before CAR-T therapy and found that only the mice treated with CPA and Nectin4 mCAR-T cells dramatically lessened tumor burden and achieved a complete remission without recurrence for more than 60 days, confirming that CAR-T therapy in combination with chemotherapy may be a promising strategy for malignant solid tumors (**Figures 4H, I**).

### Nectin4-7.19 CAR-T therapy displayed significant anti-tumor activity without on-target off-tumor toxicity for metastatic lung cancer in mice

**Figure 5:**
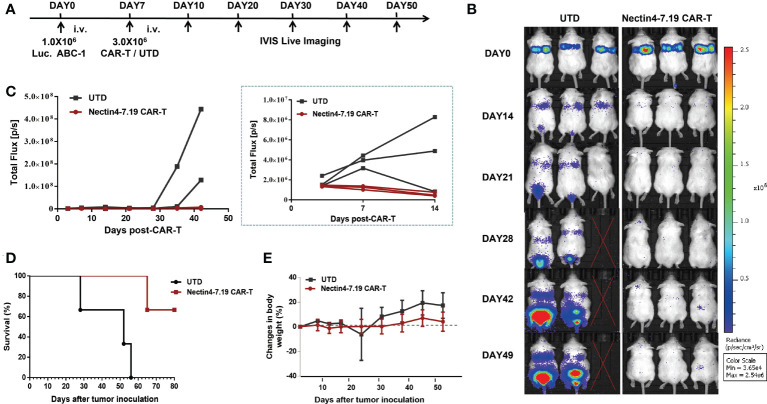
Significant anti-tumor effect of Nectin4-7.19 CAR-T therapy on metastatic lung cancer without on-target off-tumor toxicity. **(A)** NSG mice were i.v. inoculated with 1.0 × 10^6^ Luc. ABC-1 cells on Day 0 and received an administration of 3 × 10^6^ Nectin4-7.19 CAR-T cells on Day 7 (*N* = 3 mice per group). Mice treated with the same dosage of UTD cells served as a negative control. **(B–D)** Tumor xenografts were monitored *via* bioluminescence imaging. Representative bioluminescence images of three independent experiments are shown in **(B)**; bioluminescence kinetics are shown in **(C)**; solid lines represent each individual mouse. Kaplan–Meier survival curve is shown in **(D)**, *p* = 0.0246 (Nectin4-7.19 CAR-T *vs*. UTD), *N* = 3, log-rank test. **(E)** Body weight of mice since the tumor inoculation.

The severely immunodeficient mice were intravenously injected with Luc. ABC-1 cells expressing a GFP-firefly luciferase fusion protein (**Figure 3C** and **Figure S6**) and then treated with Nectin4-7.19 CAR-T cells (**Figure 5A**). Adoptive transfer with Nectin4-7.19 CAR-T cells could significantly eliminate metastases (**Figures 5B, C**), leading to a long-term survival (**Figure 5D**). However, one mouse treated with Nectin4-7.19 CAR-T cells suffered a relapse on Day 42 and finally died on Day 65.

To assess the potential on-target off-tumor toxicity of Nectin4-7.19 CAR-T therapy, we excised and examined susceptible murine organs from euthanized mice. No obvious pathological changes were detected in the organs (**Figure S7**), and no weight loss or abnormal behavior was observed in mice treated with Nectin4-7.19 CAR-T cells (**Figure 5E**).

### Combination of Nectin4-7.19 CAR-T cell therapy and FAP-12 CAR-T cell therapy showed synergistic effects in the mouse model of lung metastasis

**Figure 6:**
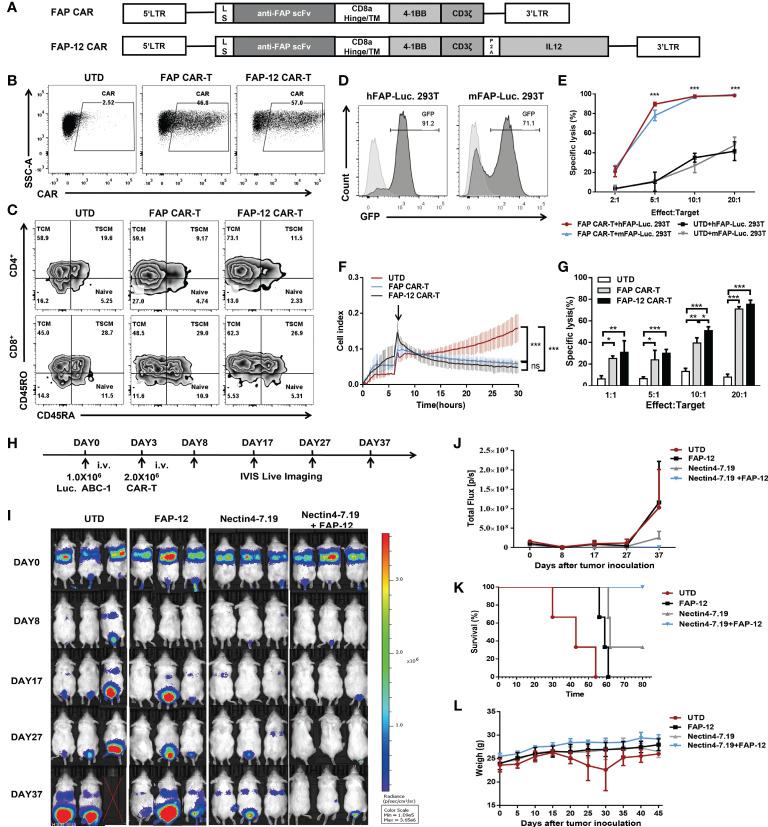
Synergistic effect of Nectin4-7.19 CAR-T with FAP-12 CAR-T therapy on metastatic lung cancer mouse model. **(A)** Schematic illustration of FAP CAR and FAP-12 CAR lentiviral vector. LS: leader signal. **(B)** CAR expression on FAP CAR-T and FAP-12 CAR-T cells. **(C)** Expression of CD45RA and CD45RO in CD4^+^ or CD8^+^ T subset to assess the subtypes of T cells. **(D)** 293T cells were transduced with lentivirus encoding the *human FAP-Firefly-Luciferase-GFP* gene or the *murine FAP-Firefly-Luciferase-GFP* gene to generate hFAP-Luc. 293T and mFAP-Luc. 293T cells, respectively. Expression of GFP was measured by flow cytometry. 293T cells served as negative controls. **(E)** Quantified data on the specific lytic levels of FAP CAR-T cells against hFAP-Luc. 293T and mFAP-Luc. 293T cells were assessed by luciferase bio-luminescence technique at different Effect/Target ratios *in vitro*. UTD served as a negative control. **(F)** Cytotoxicity of FAP CAR-T and FAP-12 CAR-T cells was detected at an Effect/Target ratio of 10:1 by xCELLigence RTCA label-free technology. **(G)** Specific lysis of FAP CAR-T and FAP-12 CAR-T cells against hFAP-Luc. 293T was detected by luciferase bio-luminescence technique at different Effect/Target ratios *in vitro*. **(H)** NSG mice were inoculated with 1.0 × 10^6^ Luc. ABC-1 cells i.v. on Day 0 and received an administration of 2 × 10^6^ FAP-12 CAR-T cells, 2 × 10^6^ Nectin4-7.19 CAR-T cells, or an admixture of 1 × 10^6^ Nectin4-7.19 CAR-T cells and 1 × 10^6^ FAP-12 CAR-T cells on Day 3 (*N* = 3 mice per group). A total of 2.0 × 10^6^ UTD served as a negative control. **(I)** Tumor xenografts were monitored *via* bioluminescence imaging. Representative bioluminescence images of three independent experiments in each group of mice were shown. **(J)** Bioluminescence kinetics of the tumor growth in the model. **(K)** Kaplan–Meier survival curve. *p*-values of log-rank tests were as follows: *p* = 0.0246 (Nectin4-7.19+FAP-12 *vs*. UTD); *p* = 0.0246 (Nectin4-7.19+FAP-12 *vs*. FAP-12); *p* = 0.1161 (Nectin4-7.19+FAP-12 *vs*. Nectin4-7.19), *N* = 3. **(L)** Body weight of mice since the tumor inoculation. Data represent the mean ± SD; *p < 0.05, **p < 0.01, ***p < 0.001, *t*-test.

To explore if FAP-12 CAR-T cells targeting CAFs could collaborate with Nectin4-7.19 CAR-T cells to enhance the anti-tumor efficacy, we constructed FAP-targeted CAR (**Figures 6A, B**) and found that there was no significant difference in phenotypic composition between FAP CAR-T and FAP-12 CAR-T cells (**Figure 6C**). Then, we generated hFAP-Luc. 293T and mFAP-Luc. 293T cells to verify the efficient cytotoxicity of FAP CAR-T cells against both murine and human FAP *in vitro* (**Figures 6D, E**) and found that FAP-12 CAR-T cells exhibited a slightly stronger specific cytotoxicity than FAP CAR-T cells (**Figures 6F, G**). In addition, we found that FAP-positive tumor stroma appeared in the ABC1 lung cancer of the NSG mouse model (**Figure S8**), and our previous study has proven the safety and effectiveness of FAP-targeted CAR-T cells in this mouse model (37).

Then, Luc. ABC-1 cells were intravenously injected into mice to establish a metastasis lung cancer mouse model (**Figure 6H**). The mice were given different therapeutic regimens (**Figure 6I**). After several weeks, the combination of Nectin4-7.19 CAR-T cells and FAP-12 CAR-T cells had the most effective anti-tumor effects (**Figure 6J**) and survival advantages compared to each monotherapy alone (**Figure 6K**). To evaluate the safety of monotherapy or combination therapy with CAR-T cells, we verified that there were no weight losses or other obvious adverse events (**Figure 6L** and **Figure S9**).

## Discussion

So far, there are more than 1,000 ongoing CAR-T therapy clinical trials, most of which are for recurrent/refractory hematological tumors. As for malignant solid tumors, an increasing number of studies have been devoted to searching for tumor-associated antigens, but only few clinical trials conducted have shown promising results, due to severe side effects and toxicities (38). Here, we described the characterization of our fourth-generation Nectin4-7.19 CAR-T and FAP-12 CAR-T cells, which were shown to possess potent proliferation, migration, and cytotoxicity *in vitro* and significant anti-tumor effect *in vivo*.

Recent reports have revealed the correlation between variations in the function of T-cell subpopulation and efficacy of CAR-T cell immunotherapy (39). T_SCM_ from a CD45RA^+^CD45RO^+^ T population expressing CCR7 and CD62L possesses higher effectiveness and persistence against tumors than T_CM_ (40). Both CD8^+^ and CD4^+^ T subsets exhibit synergistic anti-tumor CAR-T activities, as CD4^+^ cells are conducive to developing CD8^+^ memory functions (41, 42). Our data showed that expression of CAR in the CD4^+^ T subset was equal to that in the CD8^+^ subset, and the proportion of the T_SCM_ subpopulation in the CD8^+^ T subset of Nectin4-7.19 CAR-T was higher than that of Nectin4 CAR-T cells, but there was no difference between FAP CAR-T and FAP-12 CAR-T cells, which may be related to IL-7 function in retaining the subpopulation of T_SCM_ (43).

After trafficking to the tumor site and encountering their cognate antigen, T cells undergo rapid expansion to attain the appropriate numbers relative to the tumor burden. As previously reported, CCL19 could enhance recruitment and activation of CCR7-positive antigen-presenting cells and T cells by dendritic cell- and stromal cell-based intratumoral delivery (26, 30, 44), and IL-7 could stimulate proliferation of lymphocytes and maintain their survival and homeostasis (45). Furthermore, IL-7 signaling could prevent the exhaustion of T cells through a variety of mechanisms including downregulation of PD-1 expression (46). Accordingly, our Nectin4-7.19 CAR-T cells could reduce the expression of immunological checkpoints, such as PD-1, CTLA-4, TIM-3, and LAG-3, for the protection of CAR-T cells from exhaustion. Localized delivery of one or two scFvs from checkpoint blockers by CAR-T cells could enhance anti-tumor efficacy *in vivo* with minimal systematic toxicity (47). Thus, we are going to construct the fourth-generation CAR-T cells to secrete a PD-1- or/and CTLA-4-blocking scFv together with IL-7 and CCL19, which may maximize the efficiency of CAR-T therapy for malignant solid tumors.

Enfortumab vedotin (ASG-22ME), an antibody–drug conjugate targeting Nectin4, has demonstrated a clinically significant response rate with a manageable and tolerable safety profile in patients with locally advanced or metastatic urothelial carcinoma and thus received FDA approval based on phase I/II data, representing an alternative to established third-line chemotherapies with vinflunine, paclitaxel, or docetaxel (15, 48). In our study, we established Nectin4-targeted CAR-T cells based on the safety and efficacy of Nectin4 as a therapeutic target in the clinic and confirmed its capability and security *in vivo*. Nectin4 mCAR-T therapy for subcutaneous xenograft of colorectal cancer in the fully immune-competent mouse model was dose-dependent and exhibited superior anti-tumor efficacy with pretreatment of lymphodepletion. Moreover, in the highly immuno-deficient mouse model of metastatic lung cancer, Nectin4-7.19 CAR-T cells eliminated tumors effectively without inducing any obvious pathological changes in important organs or signs of graft-*vs*.-host disease. However, this may not predict an absence of toxicity in humans, since human Nectin4-targeted CAR-T cells had no cross-reaction with murine Nectin4 (24). Our phase I study (NCT03932565) addressing this issue has been ongoing to examine the safety and feasibility of Nectin4-7.19 CAR-T cells in patients with Nectin4-positive malignant solid tumors. We found that hemorrhagic rash and rash desquamation occurred due to the high expression of Nectin4 in skin tissues, but the symptoms were resolved without special treatment, and no severe CRS or neurotoxicity was observed. Therefore, Nectin4-7.19 CAR-T therapy is a promising treatment for malignant solid tumors.

Previous studies have found that cancer cells initiate and sustain the activation of CAFs while CAFs support the growth, motility, and invasion of cancer cells (49, 50). Targeting FAP with antibodies, vaccines, or pharmacological agents could lessen tumor progression in several preclinical animal models (51, 52). Nowadays, there were some preclinical studies on the use of FAP-targeted CAR-T cells to eliminate CAFs to inhibit tumor growth and enhance host immunity without serious side effects (53). A recently described CAR-T therapy was to modulate tumor stroma by CAR-T cells secreting IL-12, which was deposited in the targeted tumor lesion to attract innate immune cells toward tumor cells that were invisible to CAR-T cells (54). Hence, we engineered the FAP-12 CAR-T cells and validated their biological function *in vitro*. As tumor stroma could express murine FAP in desmoplastic human lung cancer xenografts (55), and our FAP-12 CAR-T cells could target both human and murine FAP, the combination of Nectin4-7.19 CAR-T cell therapy and FAP-12 CAR-T cell therapy for metastatic lung cancer in mice exerted a synergistic anti-tumor effect without any toxicities.

In conclusion, the delivery of Nectin4-7.19 CAR-T therapy may be a feasible strategy for Nectin4-positive malignant solid tumors. Furthermore, the combination of Nectin4-7.19 CAR-T cell therapy and FAP-12 CAR-T cell therapy will be a promising synergistic approach to co-target Nectin4-positive tumor cells and FAP-positive CAFs. However, it is necessary to further confirm the safety of this combination therapy in our phase I study due to the toxicities that may be attributed to the secreted cytokines or off-target effects.

## Data availability statement

The raw data supporting the conclusions of this article will be made available by the authors, without undue reservation.

## Ethics statement

The animal study was reviewed and approved by Laboratory Animal Ethics Committee of Wenzhou Medical University.

## Author contributions

FL, JG, YG, XJX and AZ designed this study. FL, SZ, and YH performed most of the experiments. CW, TX, XYX, TZ, LS, SK, and JZ assisted with the experiments. FL and JG analyzed and interpreted the data and wrote the manuscript. AZ, YG, and JZ assisted with the data analysis and modified the manuscript. All authors contributed to the article and approved the submitted version.

## Funding

This study was partially funded by the Scientific Research Fund of National Health Commission of China (WKJ-ZJ-1928 to JG), Guangdong Provincial Natural Science Foundation (2019A1515110831 to AZ), Wenzhou Municipal Science and Technology Research Program (ZS2017014 to JG; 2018ZY001 to AZ; Y20210153 to FL), Construction Fund of Key Medical Disciplines of Hangzhou (No. OO20200055 to YG), Sanming Project of Medicine in Shenzhen (No. SZSM201911004 to XJX), Shenzhen Science and Technology Plan Basic Research Project (No. JCYJ20180307150408596 to XJX), and Shandong Provincial Key R & D programs (2021CXGC011102 to JG).

## Acknowledgments

The authors thank volunteers for providing peripheral blood to isolate T cells.

## Conflict of interest

Author JG was employed by company Zhejiang Qixin Biotech.

The remaining authors declare that the research was conducted in the absence of any commercial or financial relationships that could be construed as a potential conflict of interest.

## Publisher’s note

All claims expressed in this article are solely those of the authors and do not necessarily represent those of their affiliated organizations, or those of the publisher, the editors and the reviewers. Any product that may be evaluated in this article, or claim that may be made by its manufacturer, is not guaranteed or endorsed by the publisher.
