# Supplementary material for: Development of Nectin4/FAP-targeted CAR-T cells secreting IL-7, CCL19, and IL-12 for malignant solid tumors
Source: Front Immunol. 2022 Nov 21;13:958082. doi: 10.3389/fimmu.2022.958082 (PMC9720259; doi:10.3389/fimmu.2022.958082)
Supplement: Supplementary file 1 [file DataSheet_1.docx]

Supplementary Material

# Supplementary Figures


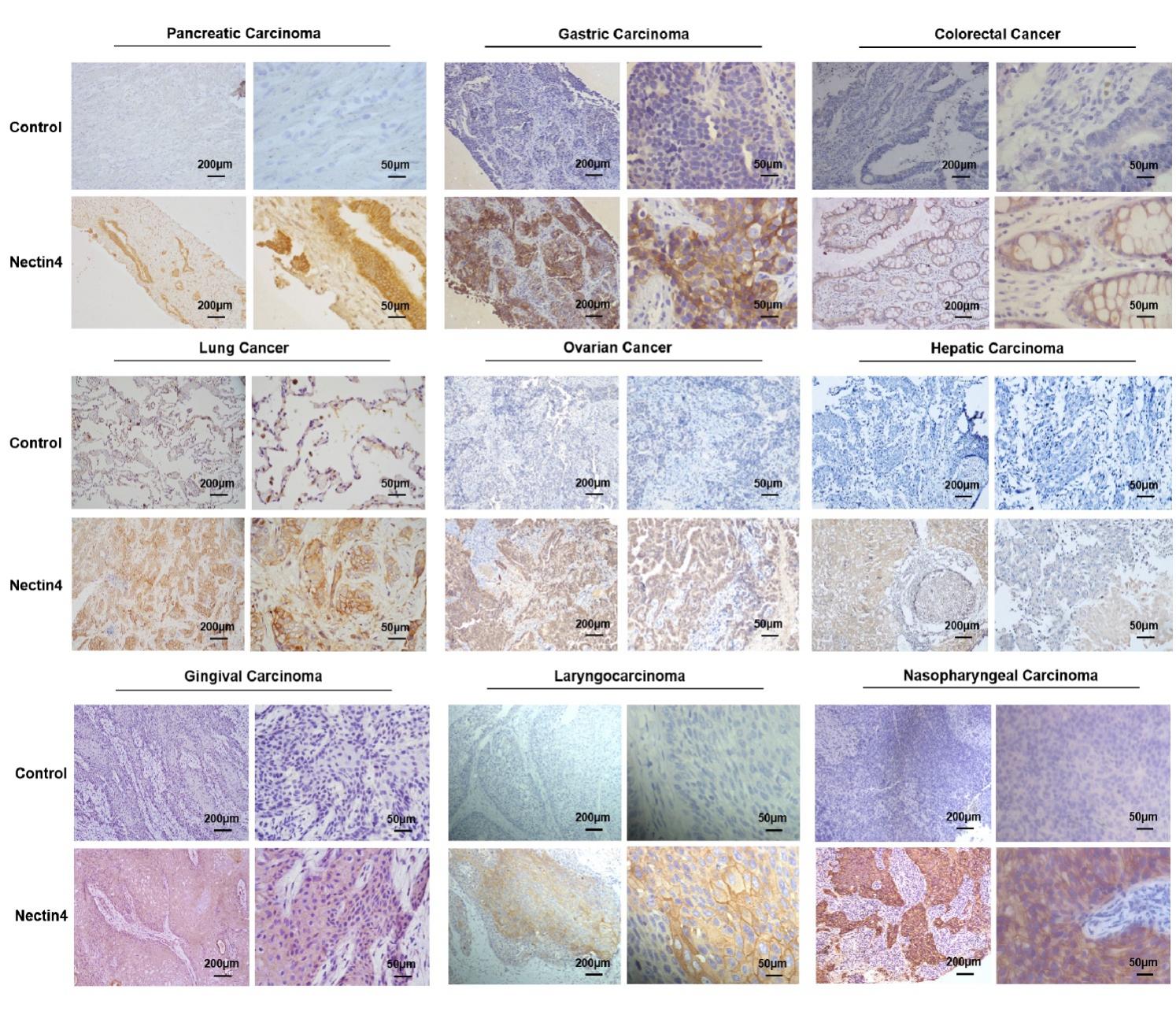


**Supplementary Figure 1.** Expression of Nectin4 in a variety of tumor biopsies was assessed by IHC. Nectin4 is mainly located in the membrane (strongly positive) and cytoplasm (weakly positive) of cancer cells, shown in brown.


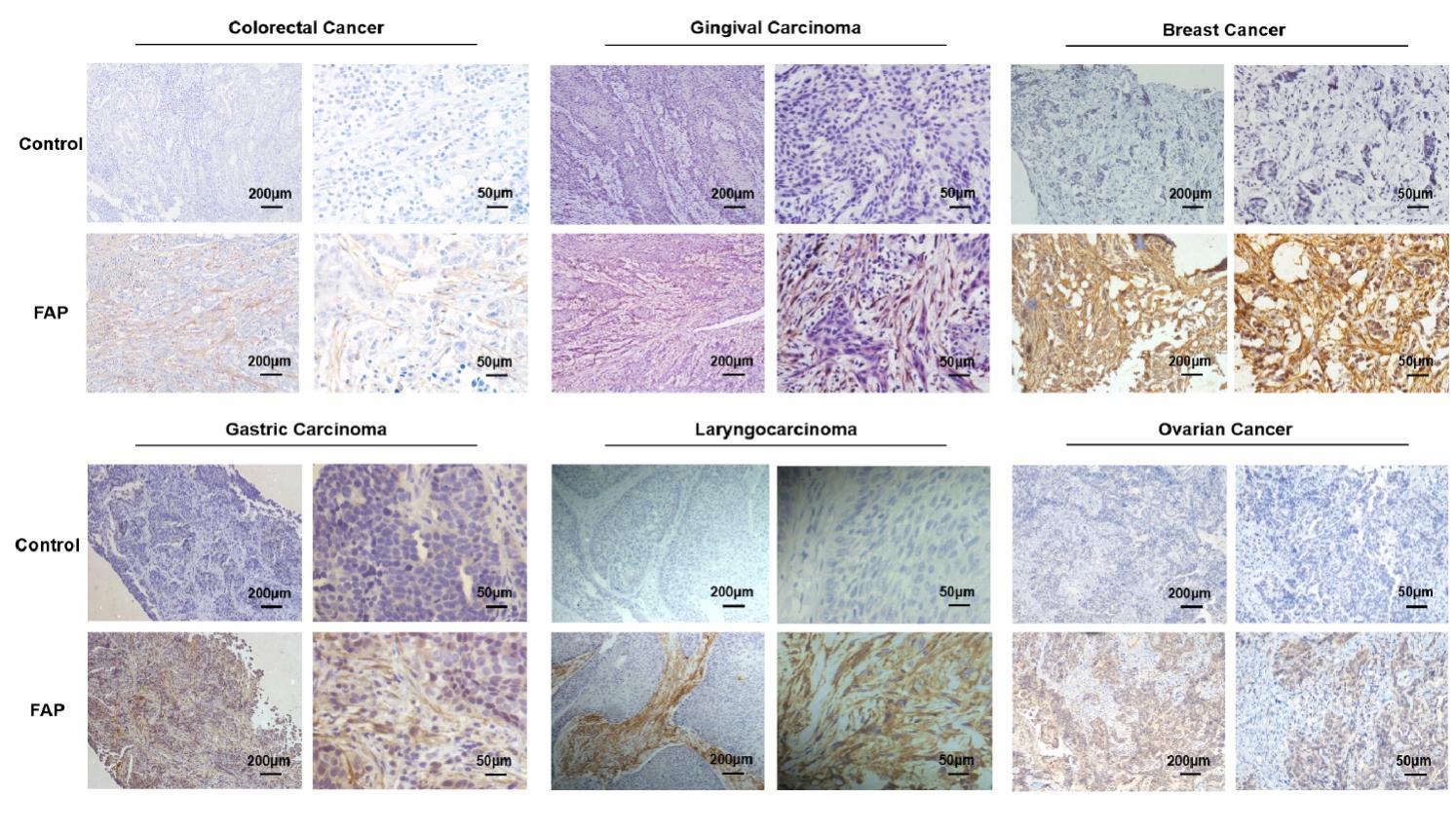


**Supplementary Figure 2.** Expression of FAP in a variety of tumor biopsies was assessed by IHC. FAP is mainly located in the membrane (strongly positive) and cytoplasm (weakly positive) of stromal cells in cancer, shown in brown.


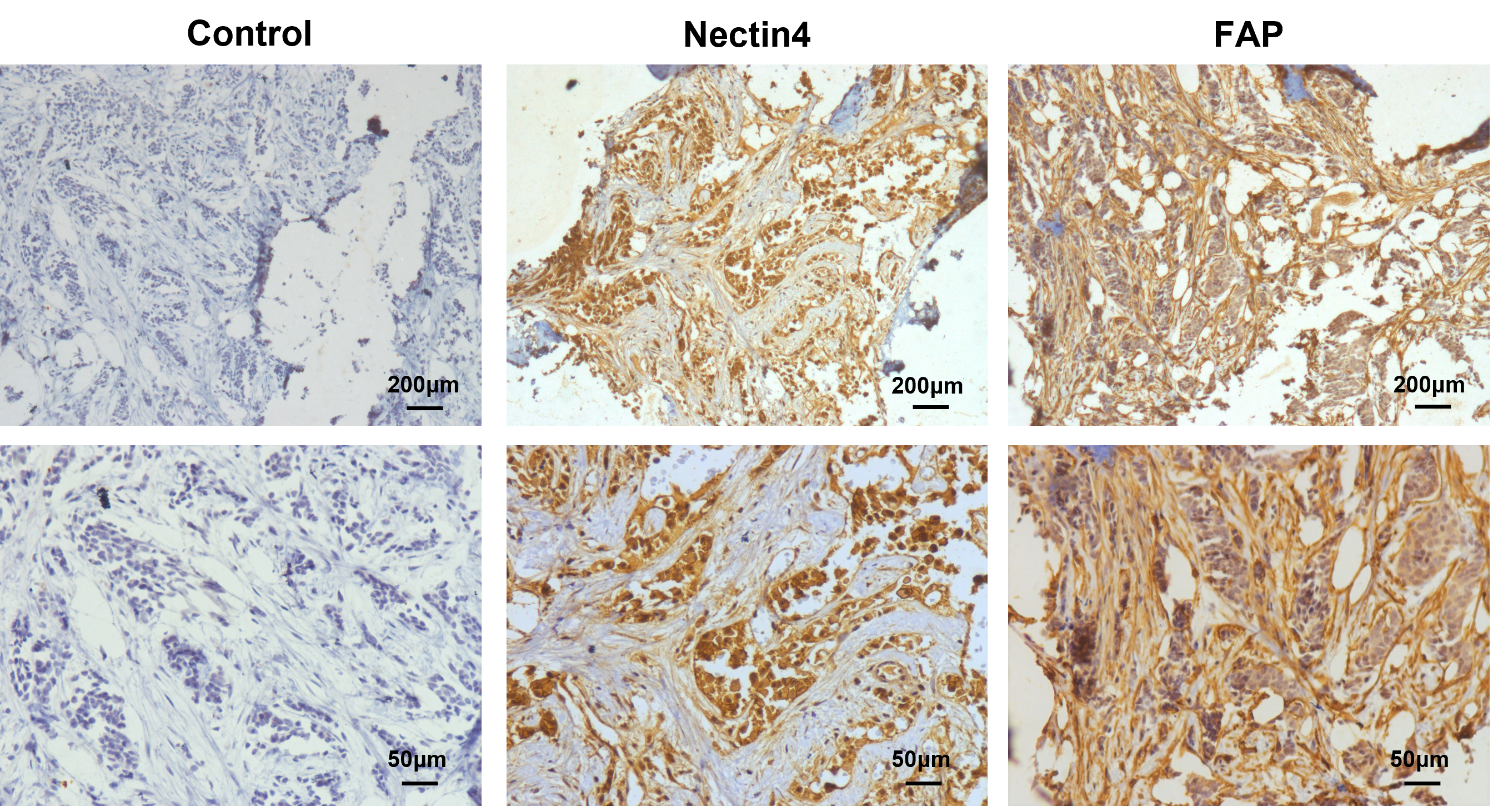


**Supplementary Figure 3.** Expression of Nectin4 and FAP on bone-metastasized triple negative breast cancer (TNBC). Nectin4 is mainly located in the membrane (strongly positive) and cytoplasm (weakly positive) of cancer cells; FAP is mainly located in the membrane (strongly positive) and cytoplasm (weakly positive) of stromal cells in cancer, shown in brown.


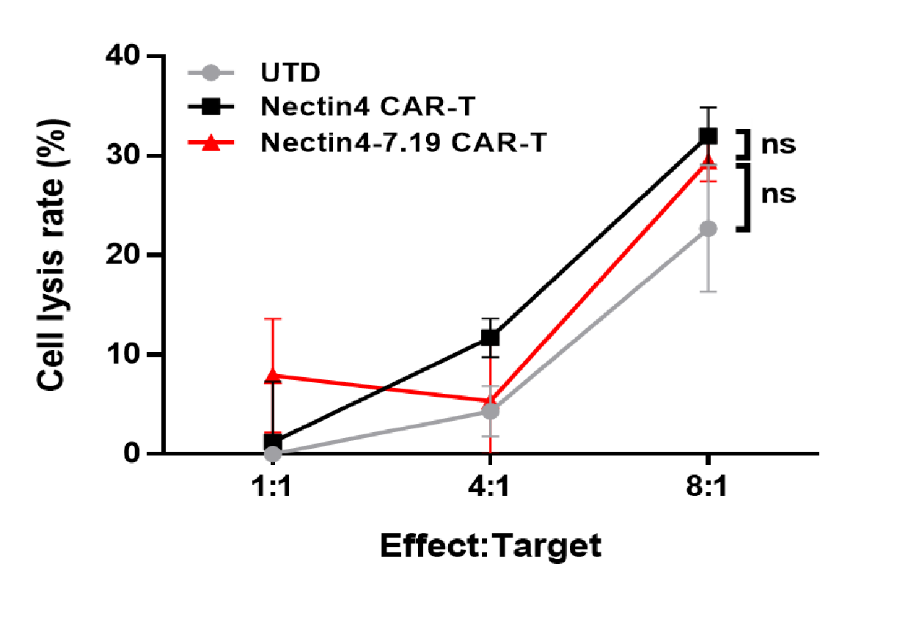


**Supplementary Figure 4.** Quantified data on the cytotoxicity of Nectin4 CAR-T and Nectin4-7.19 CAR-T cells against Luc. A549 cells were assessed by luciferase bio-luminescence technique at different Effect/Target ratios in vitro. UTD indicates the untransduced T cells served as a negative control.


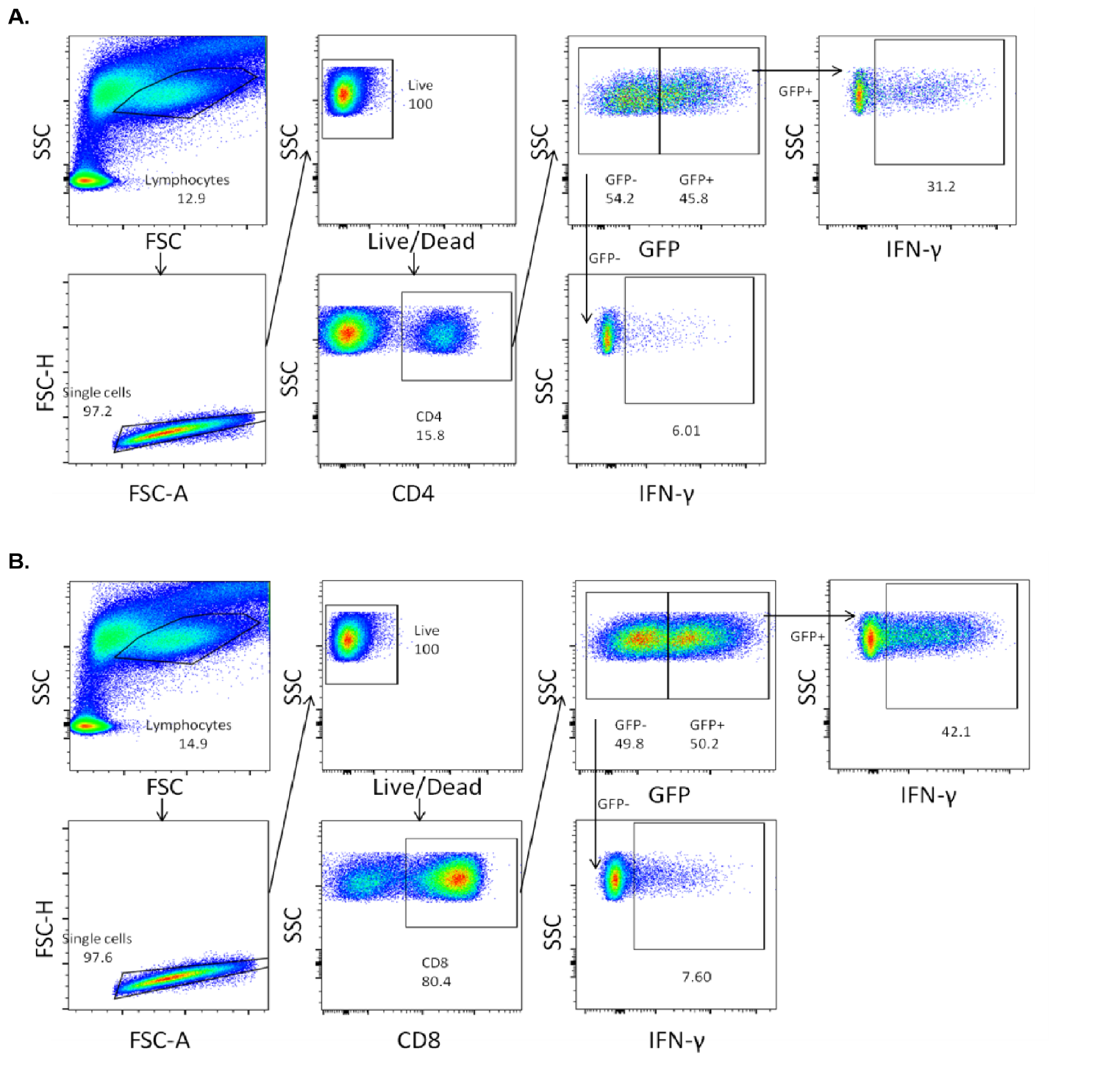


**Supplementary Figure 5.** Secretion of IFN-γ in CD4^+^ (A) or CD8^+^ (B) T subset was assayed by flow cytometry after co-culture of GFP-expressing Nectin4 mCAR-T or no-GFP-expressing mUTD cells with hNectin4-Luc. MC38 cells for 12 h. mUTD indicates the untransduced mouse T cells served as a negative control.


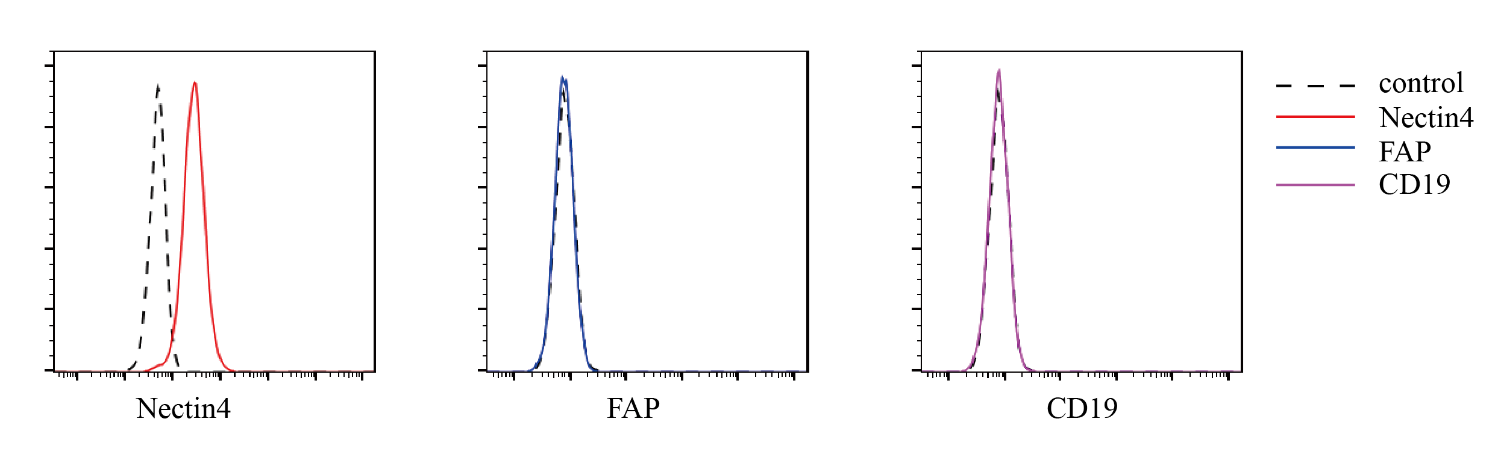


**Supplementary Figure 6.** Expression of Nectin4, FAP and CD19 on Luc. ABC1 cells. Dotted line represents the blank control represents cells stained without any antibody; red solid line represents cells stained with Nectin4 antibody; blue solid line represents cells stained with FAP antibody; purple solid line represents cells stained with CD19 antibody.


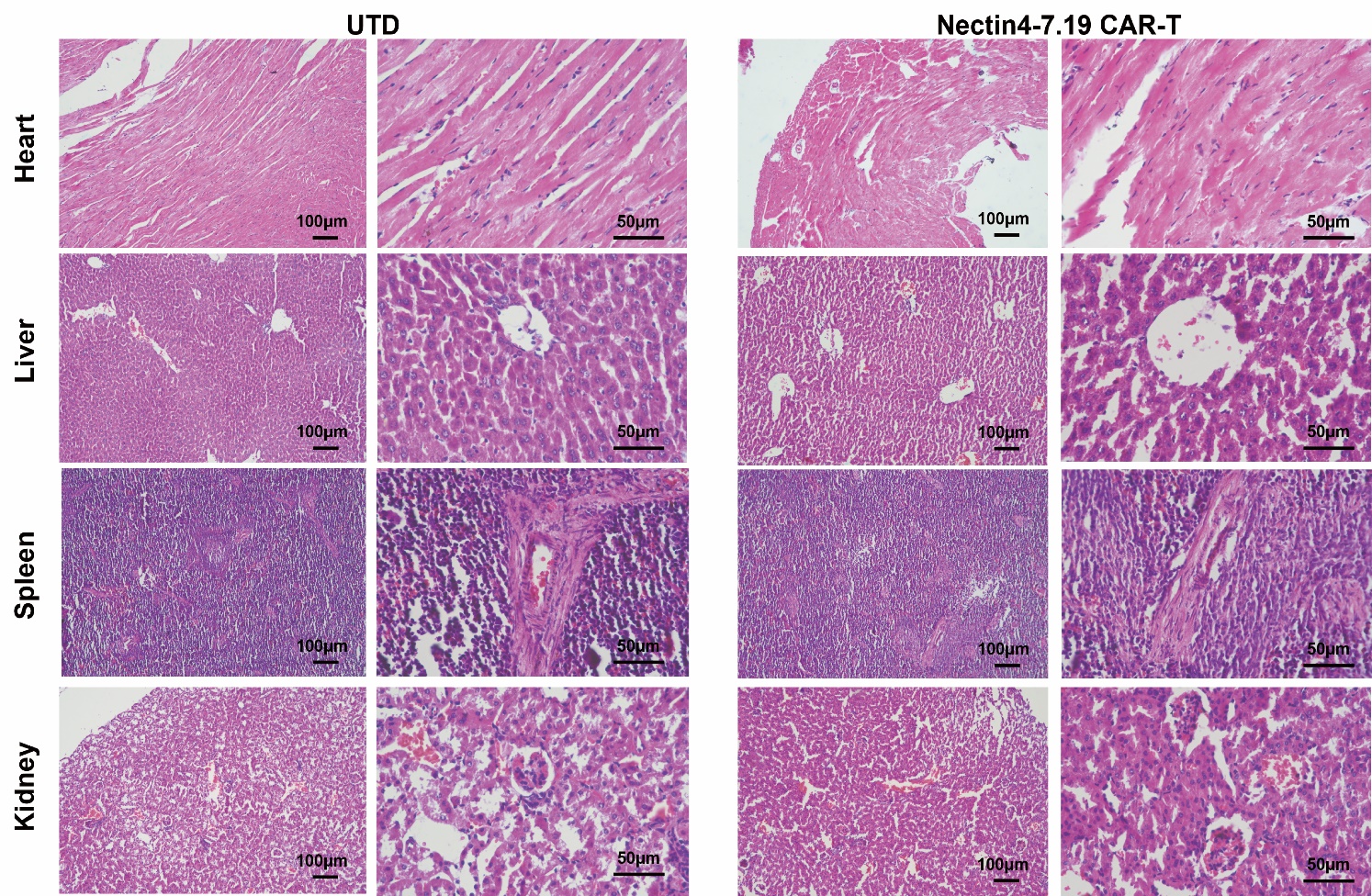


**Supplementary Figure 7.** Potential off-target toxicity of Nectin4-7.19 CAR-T therapy was evaluated via pathological changes in the organs of mice assessed by H&E staining.


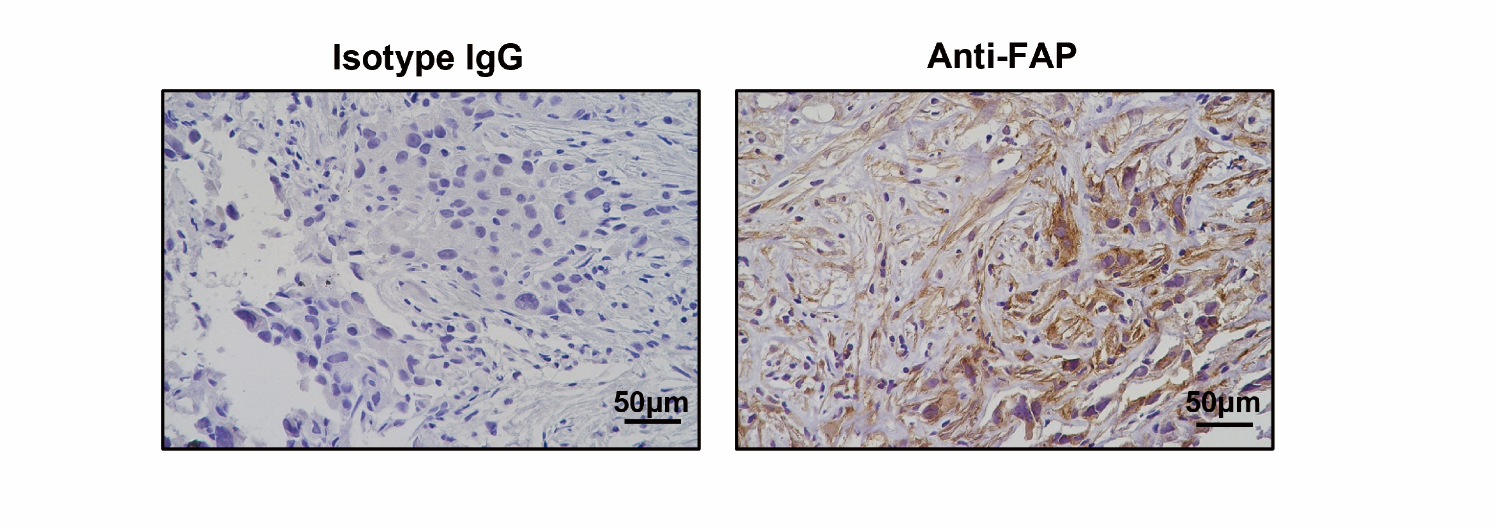


**Supplementary Figure 8.** Immunohistochemistry of ABC1 lung cancer of NSG mouse model with anti-FAP antibody. Isotype IgG served as a negative control.


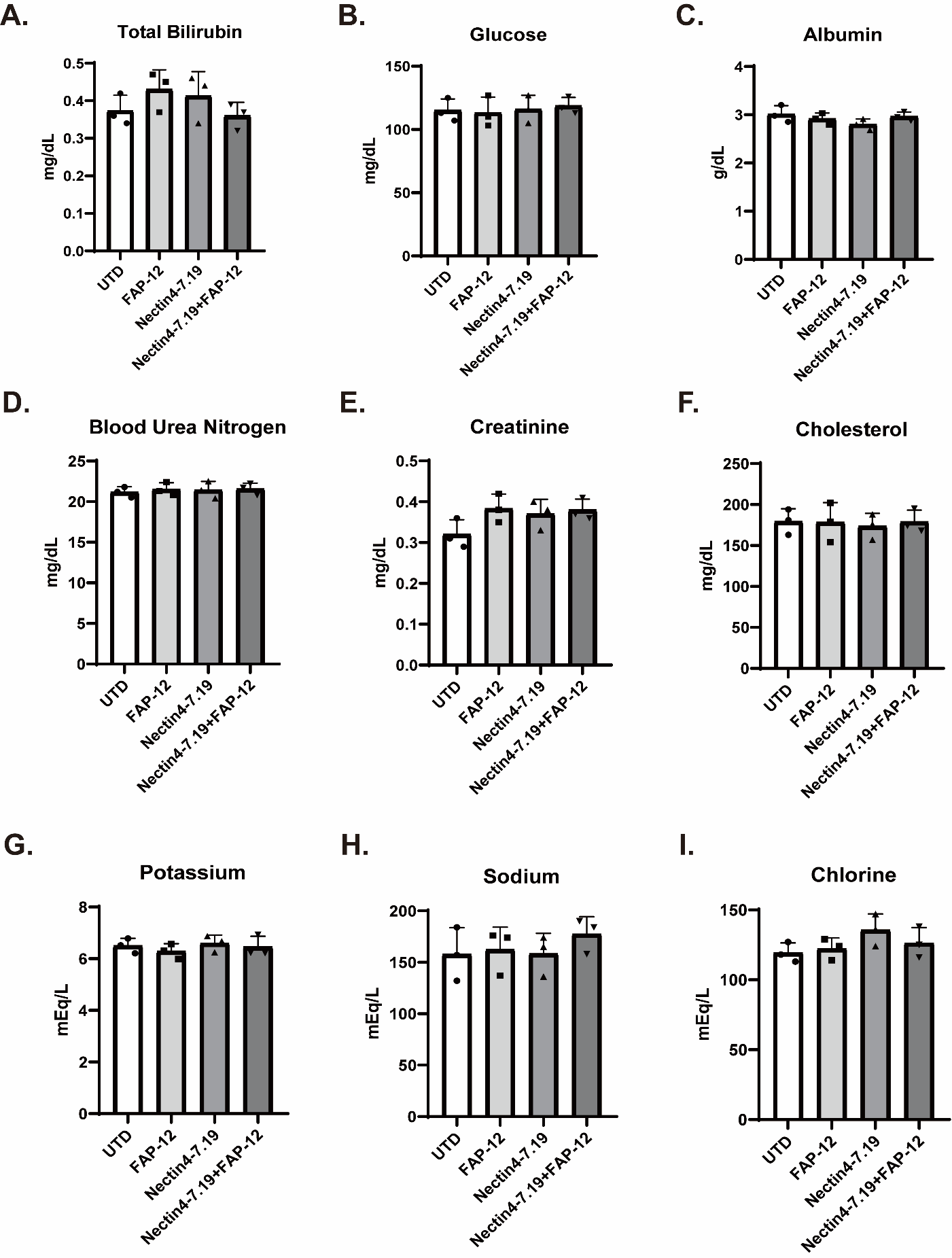


**Supplementary Figure 9.** NSG mice were inoculated with Luc. ABC-1 cells and then received an administration of different CAR-T cell therapy. Biochemical indexes were detected from the peripheral blood of mice in each group after one week of CAR-T treatment.
